# Visual outcomes of epiretinal membrane removal after diffractive-type multifocal intraocular lens implantation

**DOI:** 10.1186/s12886-022-02649-8

**Published:** 2022-11-07

**Authors:** Hyungil Kim, Sohee Jeon

**Affiliations:** 1 Gyeongju St. Mary’s Eye Clinic, Gyeongsangbuk-Do, Gyeongju-Si, Republic of Korea; 2 Keye Eye Center, Korea. 326 Teheran-Ro, Gangnam-Gu, Seoul, Korea

**Keywords:** Diffractive intraocular lens, Ectopic inner foveal layer, Epiretinal membrane, Multifocal intraocular lens

## Abstract

**Background:**

To assess visual outcomes of epiretinal membrane (ERM) removal in multifocal intraocular lens (MIOL)-implanted eyes, according to ERM stage.

**Methods:**

Retrospective chart reviews were undertaken in patients with diffractive-type MIOL implants, each undergoing pars plana vitrectomy and ERM removal between February 2018 and November 2020 at Gyeongju St. Mary's Eye Clinic and KEYE Eye Center. Assessments focused on monocular uncorrected and corrected values of distant visual acuity (UDVA and CDVA) and uncorrected near visual acuity (UNVA) at postoperative 12 months according to the stage of ERM.

**Results:**

The present study included a total of 49 MIOL-implanted eyes from 49 enrollees, 25 undergoing pars plana vitrectomy for ERM removal (11 eyes with Stage 2 and 14 eyes with Stage 3), and 24 acting as age-matched controls. There was a significant difference in UDVA and UNVA between control and Stage 3 ERM (UDVA; 0.01 ± 0.04 for control, and 0.07 ± 0.08 for stage 3 ERM, *p* = 0.035, UNVA; 0.03 ± 0.05 for control, and 0.13 ± 0.16 for Stage 3 ERM, *p* = 0.029). There were no significant differences in CDVA between groups (*p* = 0.121, ANOVA test).

**Conclusions:**

Eyes with Stage 3 ERM did not achieve visual acuity comparable to control eyes, suggesting the necessity of an early intervention for ERM in eyes with diffractive type MIOL. A meticulous preoperative retinal evaluation for ERM development is mandatory when planning diffractive-type MIOL implantation.

## Background

Given a mounting public desire for glasses-free living and improvements in modern intraocular lens (IOL) quality, implantation of multifocal IOLs (MIOLs) has increased substantially in recent decades [1]. Types of MIOL vary in optical design, but as one of the more commonly used implants, the diffractive type provides the highest power of near addition [1]. Through intentional step-wise induction of focus, there are also abrupt divisions between each zone that inevitably produce dysphotopsia and reduce contrast sensitivity (CS) [2].

A thorough preoperative corneal and retinal evaluation is key for successful MIOL implantation. Epiretinal membrane (ERM) formation is one of the most common retinal disorders, marked by varying degrees of visual symptoms [3–5]. We have found that diffractive-type IOLs are vulnerable to retinal changes. Even low-grade ERM that does not involve fovea may impact visual function in diffractive MIOL-implanted eyes [6]. Consequently, patients with MIOLs are referred to a vitreoretinal clinic for ERM removal at earlier stages of ERM development than the patients with phakic eye or monofocal IOLs.

The ideal timing of ERM removal has been an extremely controversial issue. Early surgery has a high risk–benefit ratio, with potential for recurrence, whereas late surgery limits postoperative visual recovery [7–10]. Risk–benefit determination of ERM peeling is thus more challenging in eyes with diffractive MIOLs, as the MIOL interferes with surgical view [7, 8]. Unfortunately, there is limited data on clinical outcomes of ERM removal after MIOL implantation. The present study was conducted to analyze such outcomes by stage of ERM, using normal eyes as controls.

## Methods

Retrospective chart reviews were done, aimed at patients with diffractive-type MIOL implants undergoing successful pars plana vitrectomy (PPV) and ERM removal procedures between February 2018 and November 2020 at Gyeongju St. Mary's Eye Clinic and KEYE Eye Center. Subjects who were followed for more than 12 months were included in the study. Exclusion criteria included retinal disorders other than ERM (ie, age-related macular degeneration, diabetic retinopathy, and retinal vascular occlusions), ocular trauma and prior history of any refractive or vitreoretinal surgery. This study was approved by the Institutional Review Board/Ethics Committee of KEYE Eye Center (IRB number 20200828–001). Our protocol adhered to tenets of the Declaration of Helsinki. To increase the efficiency of the study, we randomly selected an “age-matched control group” by “individual matching,” among patients with a history of diffractive-type MIOL implantation but no ERM.

**Fig. 1:**
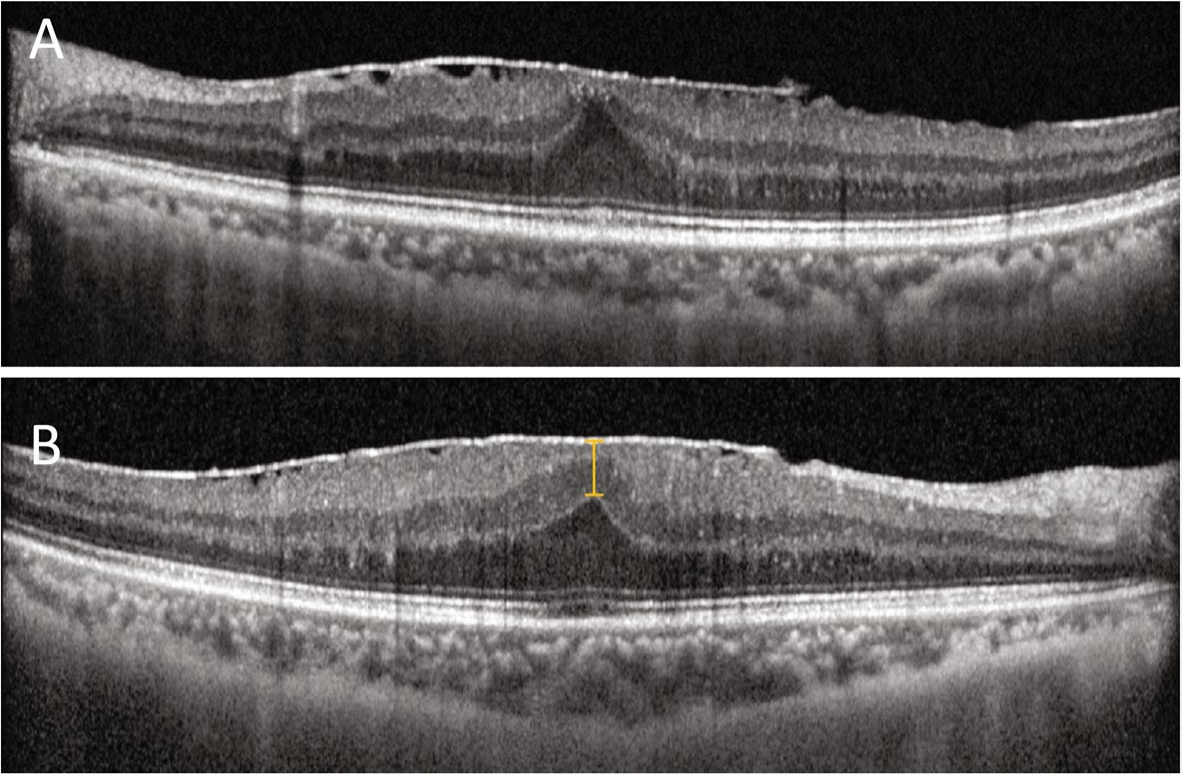
Representative images of ectopic inner foveal layers (EIFLs) at stages of epiretinal membrane (ERM) development: **A** Stage 2, loss of foveal depression but well-defined retinal layers overall; and **B** Stage 3, continuous inner nuclear (INL) and inner plexiform (IPL) layers obscuring the fovea. Yellow line indicates the thickness of EIFL

The presence and severity of ERM was determined retrospectively by spectral domain optical coherence tomography (Spectralis SD-OCT; Heidelberg Engineering, Heidelberg, Germany), based on a past study. ERM categorization was as follows: Stage 1, no anatomic distortion and preserved foveal depression; Stage 2, loss of foveal depression, but well-defined retinal layers overall (Fig. 1A); Stage 3, continuous inner nuclear (INL) and inner plexiform (IPL) layers, obscuring the fovea (Fig. 1B); and Stage 4, disruption of all retinal layers [11]. Ectopic inner foveal layer (EIFL) thickness was measured using caliper tool as the distance between inner border of outer nuclear layer (ONL) and internal limiting membrane (ILM) at foveal center by an experienced retinal specialist (S.J.) who was masked to the patient identity.

Monocular uncorrected and corrected values of distant visual acuity (UDVA and CDVA) and uncorrected near visual acuity (UNVA) were measured in decimal system, converting values to logMAR units for statistical analysis. To assess visual quality, we used two instruments (CGT-2000 [Takagi Seiko, Tokyo, Japan] and OPD-Scan III [NIDEK Co Ltd, Aichi, Japan]) to test CS at postoperative Month 12. The ocular root mean square (RMS) of higher-order aberrations (HOAs), the Strehl ratio of point spread function (PSF), and the modulation transfer function (MTF) from postoperative RMS of total ocular wave aberration Z (1 ≤ n ≤ 8) were assessed at a 5.0-mm pupil diameter. MTF was analyzed by area ratio method.

Ocular biometric findings (ie, axial length and keratometric values) were recorded by partial coherence interferometry device (IOLMaster 700; Carl Zeiss Meditec, Jena, Germany), and corneal topography (Pentacam Scheimpflug System; Oculus Inc, Wetzlar, Germany) was mapped before cataract surgery, subject to availability.

### Surgical technique

Two experienced vitreoretinal surgeons (HK and SJ) performed all operations, using a 25-gauge standard sutureless pars plana vitrectomy system (Alcon Laboratories, Geneva, Switzerland). The NGENUITY 3D Visualization System (Alcon Laboratories) was used at the KEYE Eye Center. Subtenon anesthesia (lidocaine) was applied prior to surgery. The trocar was placed ~ 3.5 mm posterior to limbus in three quadrants: superotemporal, inferotemporal, and superonasal. Once core vitrectomy was achieved (as needed), the posterior hyaloid membrane was detached by utilizing the vitrectomy probe in suction mode around optic nerve disc. Peripheral vitreous shaving was then conducted under great scrutiny, moving clockwise at hourly positions. The ERM was peeled away by intraocular forceps coated with triamcinolone (MaQaid; Hanmi Pharmaceutical Co Ltd, Seoul, Korea). ILM was removed at the discretion of the surgeon, either concurrently or after ERM removal within a fovea-centered circular area of 2–3 optic disc diameters using 0.5% indocyanine green (ICG) dye (Dongindang Inc., Seoul, Korea). The FINESSE Flex Loop (Alcon Laboratories) was engaged if iatrogenic retinal damage was suspected after ERM or ILM manipulation, owing to blurred surgical field. Finally, the periphery was closely inspected to ensure its integrity (absence of retinal holes or tears). No intraocular tamponade was used in these cases. There were no periocular injections of antibiotics or steroids. Postoperatively, topical moxifloxacin 5 mg/mL (Vigamox; Novartis AG, Basel, Switzerland) and prednisone acetate 1% (Pred Forte; Allergan TechAlliance, Dublin, Ireland) were applied four times daily for 4 weeks.

### Statistical analysis

All computations were driven by standard software (SPSS v15.0 for Windows; SPSS Inc, Chicago, IL, USA). Descriptive data were expressed as mean ± standard deviation values, unless otherwise specified. The Shapiro–Wilk test served to assess normality of continuous variables, using analysis of variance (ANOVA) to compare three or more data points and invoking Bonferroni test for *post-hoc* analysis. The comparisons of paired variables were calculated with Wilcoxon signed test and the comparisons of non-paired variables were calculated with Mann–Whitney test. All *p* values were two-sided, setting significance at < 0.05. The minimum sample size of 9 eyes for each group was calculated by G*Power3 software (Dusseldorf, Germany) with a significance level (α) of 0.05, power of 0.90, effect size of 1.256 which was calculated based on our preliminary data for comparison of CDVA at baseline and postoperative 12 month.

## Results

The present study included a total of 49 eyes from 49 enrollees, 25 undergoing to pars plana vitrectomy for ERM removal and 24 acting as age-matched controls. Baseline demographics of test and control groups are summarized in Table 1. Mean age was similar for both groups (ERM, 63.92 ± 5.27 years; controls, 63.63 ± 3.89 years; *p* = 0.809). There were six men (24.0%) in the ERM group and 11 (45.8%) in the control group (*p* = 0.140). Mean follow-up periods after ERM (18.68 ± 8.88 months) and after cataract surgery (18.96 ± 5.96 months) in controls were not significantly different (*p* = 0.898). There was no difference in the presence of diabetes (8.0% for ERM group and 8.3% for control group; *p* = 0.680) or hypertension (44.0% for ERM group and 41.7% for control group; *p* = 0.549) between groups. No difference was detected in the previous LASER photocoagulation (12.0% for ERM group and 8.3% for control group; *p* = 0.520), refractive error (-0.48 ± 0.66 D for ERM group and -0.38 ± 0.26 D for control group; *p* = 0.283), or refractive astigmatism (-0.57 ± 0.34 D for ERM group and -0.47 ± 0.26 D for control group; *p* = 0.510). ERM group showed significantly higher central subfield thickness (CST; 398.85 ± 53.44 µm) when compared with the control group (266.25 ± 23.38 µm; *p* < 0001). There was no eye with macular edema or metamorphopsia before or after ERM removal; and no major related complications, such as retinal detachment, endophthalmitis, vitreous hemorrhage, or hypotony, were encountered postoperatively.

**Table 1:** Clinical characteristics of enrolled patients

Characteristics	ERM group (*n* = 25)	Control group (*n* = 24)	*P* value
Age, years	63.92 ± 5.27	63.63 ± 3.89	0.809
Follow up, months	18.68 ± 8.88	18.96 ± 5.96	0.898
Sex, male (%)	6 (24.0)	11 (45.8)	0.140
Diabetes, yes (%)	2 (8.0)	2 (8.3)	0.680
Hypertenstion, yes (%)	11 (44.0)	10 (41.7)	0.549
Laterality, OD (%)	15 (60.0)	15 (62.5)	> 0.999
Previous LASER photocoagulation, yes (%)	3 (12.0)	2 (8.3)	0.520
Refractive error in SE, D	-0.48 ± 0.66	-0.38 ± 0.26	0.283
Refractive astigmatism, D	-0.57 ± 0.34	-0.47 ± 0.26	0.510
Axial lengths, mm	23.53 ± 0.70	23.86 ± 0.98	0.285
Mean keratometry, D	44.09 ± 1.76	43.96 ± 1.26	0.796
Corneal astigmatism, D	-0.61 ± 0.38	-0.58 ± 0.26	0.762
Central subfield thickness, µm	398.85 ± 53.44	266.25 ± 23.38	< 0.001
Grade of ERM			
Grade 2, no (%)	11 (44.0)		
Grade 3, no (%)	14 (56.0)		

Data are mean ± standard deviation (range) unless otherwise indicated

*EIFL* ectopic inner foveal layer, *ERM* epiretinal membrane, *SE* spherical equivalent

**Fig. 2:**
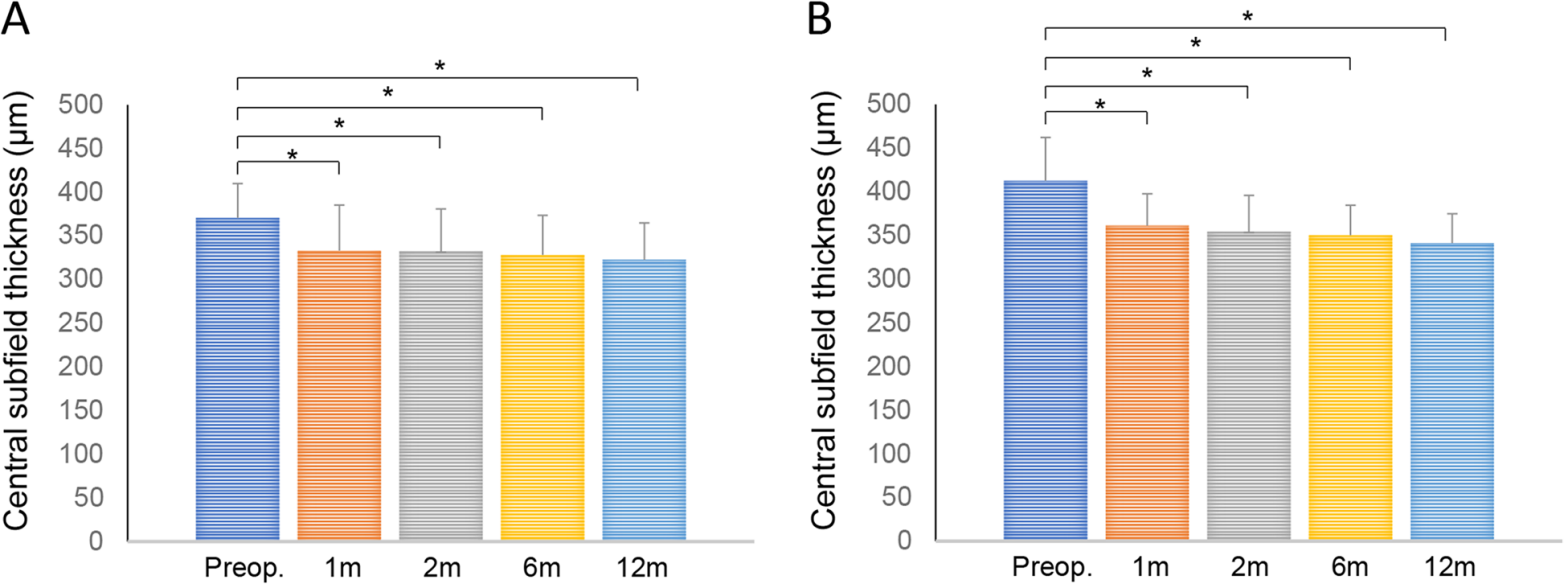
Changes in central subfield thickness after epiretinal membrane removal in Stage 2 (**A**) and Stage 3 (**B**; *p < 0.05)

**Fig. 3:**
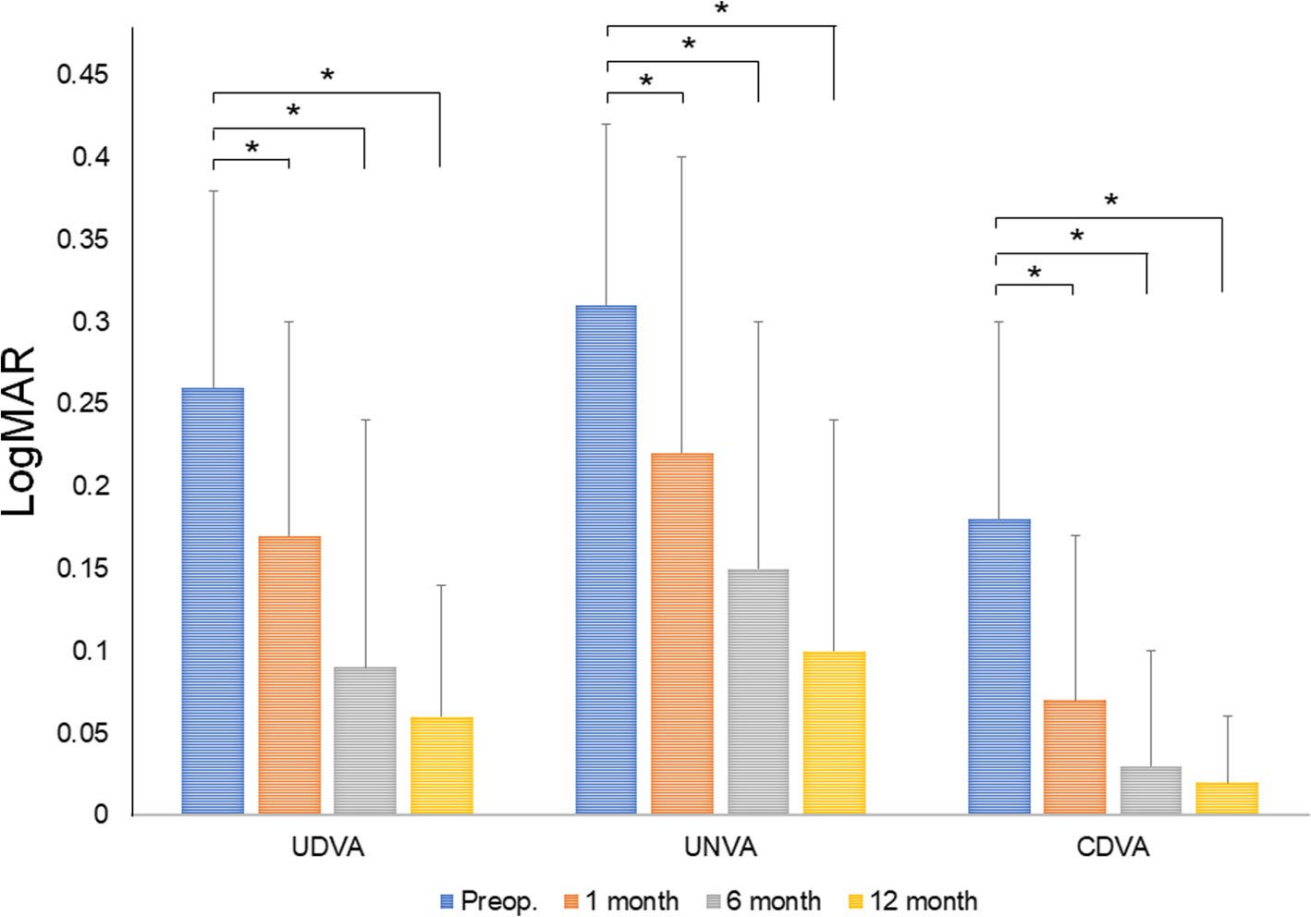
Changes in visual acuity after epiretinal membrane removal. CDVA, corrected distant visual acuity; UDVA, uncorrected distant visual acuity; UNVA, uncorrected near visual acuity (*p < 0.05)

At time of ERM removal, 11 of 25 eyes (44.0%) showed Stage 2 ERM, the remaining 14 (56.0%) qualifying as Stage 3. Mean EIFL thickness in eyes with Stage 3 ERM was 103.45 ± 53.52 µm (range, 22.0–198.0 µm). Figure 2 underscores changes in CST before and after ERM removal. CST improved significantly over time in both stage of ERM (Stage 2, 370.45 ± 39.38 µm at baseline; 333.00 ± 52.14 µm at 1 month, *p* = 0.006; 332.18 ± 48.66 µm at 2 month, *p* = 0.008; 327.91 ± 45.42 µm at 6 month, *p* = 0.016; 322.45 ± 42.21 µm at 12 month, *p* = 0.008*:* Stage 3, 412.57 ± 49.66 µm at baseline; 361.21 ± 36.43 µm at 1 month, *p* = 0.002; 354.17 ± 41.63 µm at 2 month, *p* = 0.002; 350.00 ± 34.49 µm at 6 month, *p* = 0.001; 341.07 ± 33.83 µm at 12 month, *p* = 0.001*)*. CDVA, UDVA, and UNVA also improved significantly at each time point (CDVA: *p* = 0.007, *p* < 0.001, and *p* < 0.001; UDVA: *p* < 0.001, each; UNVA: *p* < 0.001, each) (Fig. 3).

**Fig. 4:**
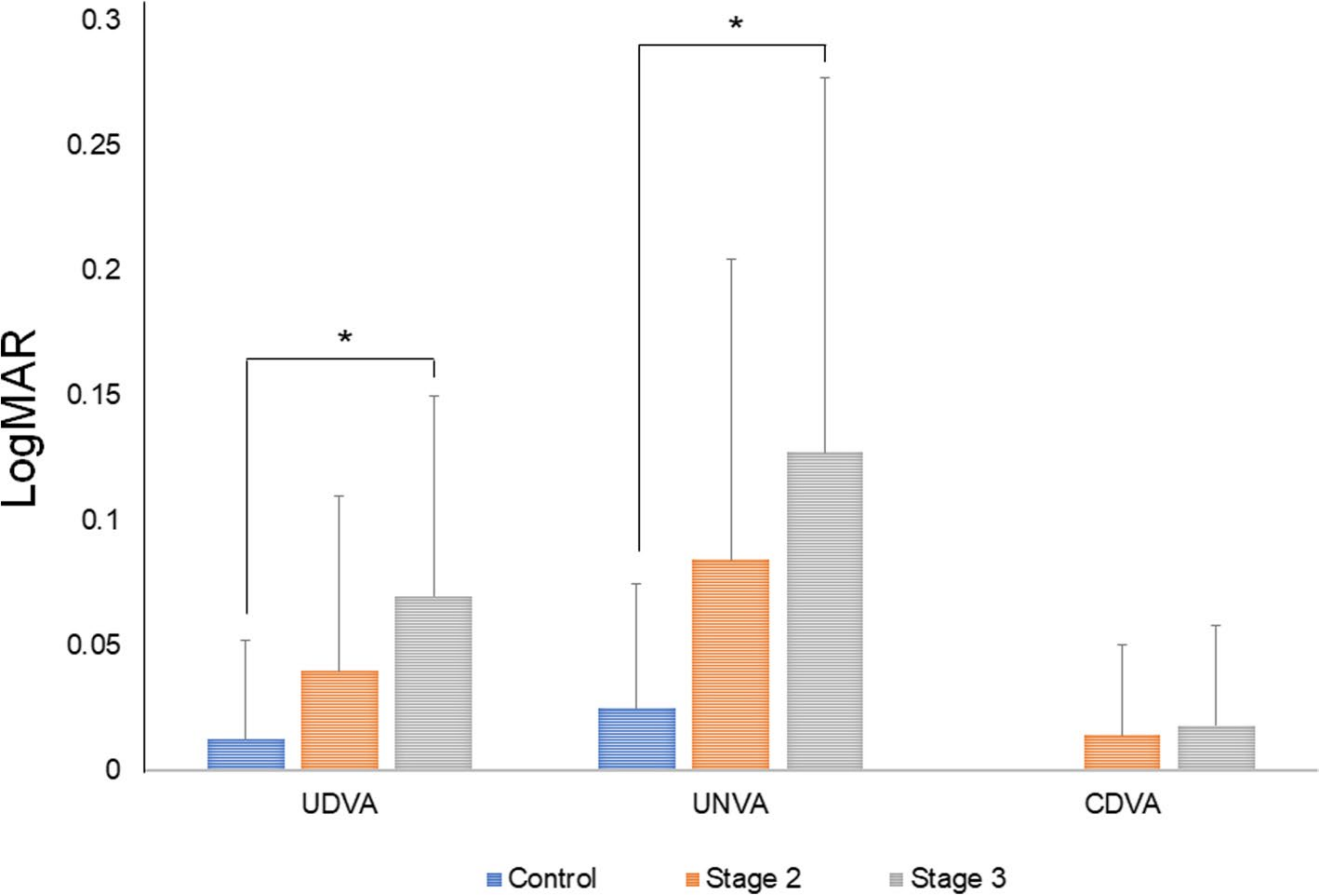
Comparison of visual acuity after epiretinal membrane (ERM) removal in ERM subgroups according to the stage of ectopic inner foveal layer (EIFL). CDVA, corrected distant visual acuity; EIFL, ectopic inner foveal layer; UDVA, uncorrected distant visual acuity; UNVA, uncorrected near visual acuity (*p < 0.05)

In Fig. 4, visual outcomes of MIOL implanted eyes are documented by retinal status. Some differences in UDVA (*p* = 0.035, ANOVA test) and UNVA (*p* = 0.025, ANOVA test) were significant, but CDVA did not differ significantly between groups (*p* = 0.121, ANOVA test). UDVA of Stage 2 (0.04 ± 0.07), Stage 3 (0.07 ± 0.08), and control (0.01 ± 0.04) groups differed significantly only when comparing Stage 3 and control groups (Stage 2 vs controls, *p* = 0.475; Stage 3 vs controls, *p* = 0.035; Stage 2 vs Stage 3, *p* = 0.735 [Bonferroni test]). The same was true for UNVA of Stage 2 (0.08 ± 0.12), Stage 3 (0.13 ± 0.16), and control (0.03 ± 0.05) groups (Stage 2 vs controls, *p* = 0.306, Stage 3 vs controls, *p* = 0.029; Stage 2 vs Stage 3, *p* = 0.936 [Bonferroni test]). Thus, the Stage 3 group significantly underperformed control subjects in terms of UDVA and UNVA. There were no significant differences in UDVA or UNVA when comparing the Stage 2 group with either the control or the Stage 3 group. However, CDVA of Stage 2 (0.01 ± 0.04), Stage 3 (0.02 ± 0.04), and control (0.00 ± 0.00) groups displayed no significant differences (Stage 2 vs controls, *p* = 0.367; Stage 3 vs controls, *p* = 0.212; Stage 2 vs Stage 3, *p* > 0.999 [Bonferroni test]).

**Fig. 5:**
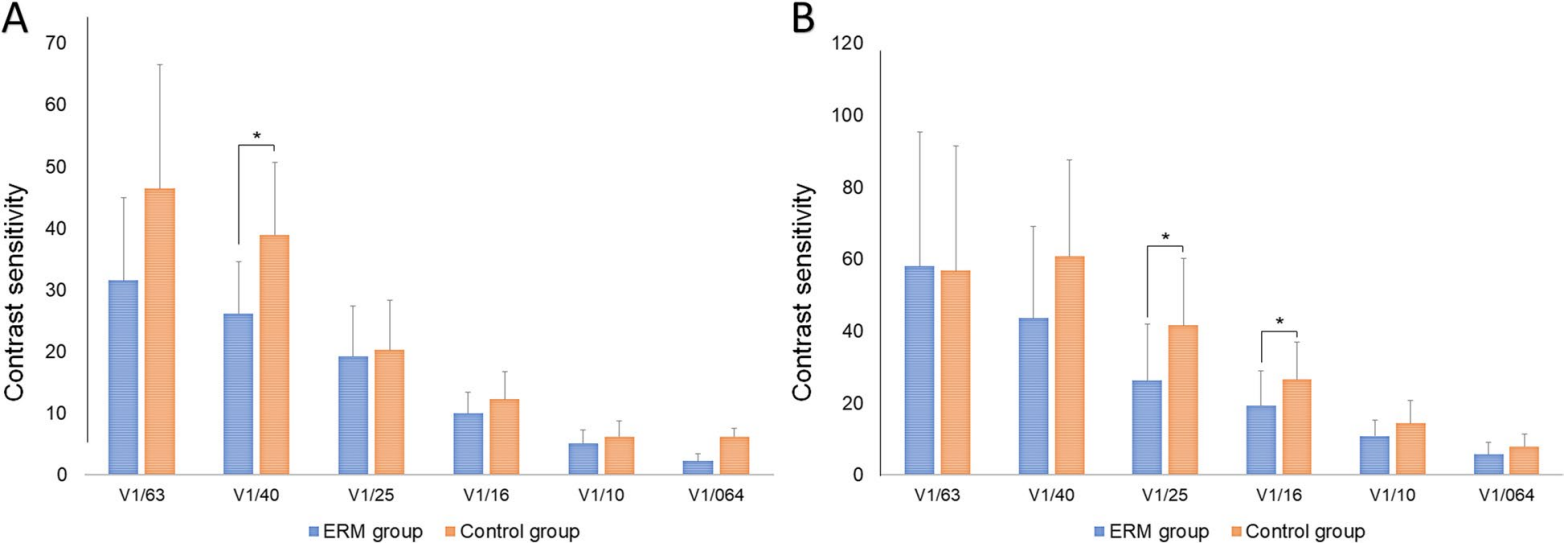
Comparison of contrast sensitivity under **A** mesopic and **B** photopic conditions in treated epiretinal membrane (ERM) and control groups (*p < 0.05)

CS proved significantly lower in the ERM (vs control) group under scotopic conditions at 4.0˚*(p* = 0.041) and at 2.5˚ (*p* = 0.014) or 1.6˚ (*p* = 0.036) under photopic conditions (Fig. 5). Strehl ratios and area ratios (4 mm and 5 mm, respectively) assessed by OPD-scan showed no significant group-wise differences (*p* = 0.162, *p* = 0.131, and *p* = 0.060, respectively; Table 2).

**Table 2:** Objective quality of vision detected by OPD-scan

Characteristics	ERM group (*n* = 25)	Control group (*n* = 24)	*P* value
Strehl ratio	0.03 ± 0.02	0.04 ± 0.02	0.162
Area ratio, 4 mm	41.80 ± 10.73	46.99 ± 11.02	0.131
Area ratio, 5 mm	45.80 ± 13.39	53.43 ± 12.41	0.060

*ERM* epiretinal membrane

## Discussion

In the present study, we found that PPV and ERM removal significantly improved visual acuity at 12 months in MIOL-implanted eyes with ERM that showed Stage 2 or 3 ERM. At postoperative month 12, visual acuity achieved in the Stage 2 group was comparable to that of control subjects, although this did not hold true for the Stage 3 group. We suspect that disorganized inner retinal structures in the Stage 3 group served to limit visual recovery, even after successful ERM removal.

Theoretically, a perfect optical system allows the focusing of light rays at a single point. In eyes with diffractive-type MIOLs, light is diffracted at IOL plane and focused in multiple points at retinal plane [1]. In Stage 2 ERM, light penetration would be marginally diminished at the ERM plane, given the opacity caused by various cells and extracellular matrix [12]. When progressing to Stage 3, diffracted light at IOL plane is further diffracted as it penetrates an irregular inner retinal structure, creating substantial visual disturbance. Despite successful ERM removal, such inner retinal changes cannot be readily reversed. Our data indicate that after ERM removal, the Stage 3 group failed to achieve a level of visual acuity comparable to that of control subjects.

The surgical timing for ERM has always been controversial, given the obstacles to precise risk–benefit ratio calculation. First, there is no standard method for predicting visual outcomes after ERM removal. Furthermore, the risk of surgical complications largely depends on level of surgical skill and a patient’s vitreoretinal status. Finally, one cannot reliably predict ERM progression at the early stage, because oftentimes ERM does not rapidly evolve.

Younger patients are increasingly undergoing cataract surgery, and as the number of MIOL implants rises, the risk of postoperative ERM development is heightened consequently. Previously, we have found that an abnormal vitreoretinal interface carries a significantly greater risk of ERM occurrence after cataract surgery, having analyzed the risk ratios of several imaging parameters [13]. In at-risk eyes, selecting an IOL with lower dysphotopsia and higher CS propensities than a diffractive-type MIOL may ultimately help avoid unnecessary vitrectomy for early ERM removal. A large-scale prospective study, including longer observation periods, is essential to determine the actual risk–benefit ratio of early ERM intervention.

## Conclusions

ERM removal significantly improved visual acuity in MIOL-implanted eyes with ERM that showed Stage 2 or 3 ERM. But the eyes with Stage 3 ERM did not achieve visual acuity comparable to control eyes, suggesting the necessity of an early intervention for ERM in eyes with diffractive type MIOL. A meticulous preoperative retinal evaluation for ERM development is mandatory when planning diffractive-type MIOL implantation.

## Data Availability

The datasets generated and analyzed during the current study are available from the corresponding author on reasonable request.

## Abbreviations

CST: Central subfield thickness
CS: Contrast sensitivity
CDVA: Corrected distant visual acuity
EIFL: Ectopic inner foveal layer
ERM: Epiretinal membrane
HOAs: Higher-order aberrations
INL: Inner nuclear layer
IPL: Inner plexiform layers
ILM: Internal limiting membrane
IOL: Intraocular lens
MTF: Modulation transfer function
MIOLs: Multifocal IOLs
ONL: Outer nuclear layer
PPV: Pars plana vitrectomy
PSF: Point spread function
RMS: Root mean square
UDVA: Uncorrected distant visual acuity
UNVA: Uncorrected near visual acuity
